# Effect of Tongue-Hold Swallow on Laryngeal Elevation During Swallowing in Healthy Older Men

**DOI:** 10.7759/cureus.68245

**Published:** 2024-08-30

**Authors:** Fumitaka Omori, Masako Fujiu-Kurachi, Aya Hirata, Takafumi Yamano

**Affiliations:** 1 Department of Otorhinolaryngology, Fukuoka Dental College Hospital, Fukuoka, JPN; 2 Department of Speech, Language and Hearing Sciences, International University of Health and Welfare, Narita, JPN; 3 Department of Speech, Language and Hearing Sciences, International University of Health and Welfare, Otawara, JPN; 4 Section of Otorhinolaryngology, Department of Medicine, Fukuoka Dental College, Fukuoka, JPN

**Keywords:** surface electromyography, healthy adults, laryngeal elevation measurement device, laryngeal elevation, tongue-hold swallow

## Abstract

Objective: This study aimed to determine whether tongue-hold swallow (THS) training affects laryngeal elevation in healthy older men during swallowing and whether THS induces muscle activity comparable to that of the Mendelsohn maneuver (MM), which is known to improve laryngeal elevation.

Methods: In study 1, 10 healthy older men were trained (two sets/day, five days/week for six weeks) with THS. They visited the clinic four times to evaluate the training effect with the maximum tongue pressure as well as the distance and peak velocity of laryngeal elevation. Laryngeal elevation was measured in a total of four conditions, two bolus conditions (saliva and water) and two swallowing methods (normal swallow (NS) and effortful swallow (ES)), using a noninvasive laryngeal elevation measurement device. In study 2, surface electromyography was performed on the same participants as in study 1 to measure integrated electromyography (iEMG) and iEMG/s during NS, THS, and MM by placing the surface electrodes on the submental muscle region and the thyrohyoid muscle region.

Results: In study 1, the maximum tongue pressure was significantly different between the first and other three evaluation visits and between the second and fourth visits, increasing gradually. Regarding the laryngeal elevation, only the distance showed a statistically significant increase between the first and fourth visits in the saliva/ES condition. In study 2, the iEMG/s of the submental muscles was greater in THS than in NS, while the iEMG value was greater in THS and MM than in NS. The iEMG value of the thyrohyoid muscle region was greater in MM than in NS and also in MM than in THS.

Conclusions: The maximum tongue pressure and laryngeal elevation distance in the saliva/ES condition were greater after the training. THS and MM showed similar levels of muscle activity in the submental muscles but not in the thyrohyoid muscle region. Therefore, the combination of THS with other training is recommended in strengthening the laryngeal elevation. THS may contribute not only to an increase of the posterior pharyngeal wall constriction and tongue retraction but also to an enhancement of the laryngeal elevation.

## Introduction

Tongue-hold swallow (THS) is a dry swallow exercise in which the tongue is protruded forward and held between the anterior teeth. This training technique has been proposed to increase the posterior pharyngeal bulging in response to insufficient contact between the tongue base and the posterior pharyngeal wall [1]. It was also suggested to enhance the tongue to move backward [2].

To date, a number of studies have attempted to elucidate the kinetic mechanisms of THS. During THS, the magnitude and duration of tongue and pharyngeal constrictor muscle activity increased [3], the duration and amount of submental muscle activity increased concurrently [4], and tongue pressure generation increased at the posterior circumferential of the hard palate [5]. In addition, tongue base-pharyngeal wall pressures and contact duration increased [6], and mesopharyngeal contraction prolonged [7]. Regarding the effect on hyoid and laryngeal elevation, Aihara et al. [8] performed a three-dimensional kinematic analysis using a 320-row area detector computed tomography (CT) and reported that the upward distance of the larynx was greater with THS than that during saliva swallowing.

To our knowledge, only a few studies have used THS as a training technique. Kimura et al. [9] reported that a patient who presented with insufficient contact between the tongue base and the pharyngeal wall underwent therapy with THS alone for three months and the insufficiency of the contact improved. Lee et al. [10] randomized older patients to three groups (THS group, tongue pressure resistance training group, and control group) and found that the THS group exhibited improvement in anterior and posterior tongue muscle strength.

Sunada et al. [11] reported that exercise with 100% maximum tongue pressure resulted in greater iEMG of submental and thyrohyoid muscle regions than at 80%. Since laryngeal elevation is influenced by submental and thyrohyoid muscles, increased tongue pressure may contribute to improved laryngeal elevation.

Although THS has been shown to increase the submental muscle activity and distance of the upward movement of the larynx during the execution of the maneuver, it is unclear whether laryngeal elevation improves after THS therapy.

The Mendelssohn maneuver (MM) is one of the swallowing maneuvers, a technique that voluntarily adjusts laryngeal elevation during swallowing to increase the amount and duration of laryngeal elevation. Furthermore, MM, with continued practice, is considered a muscle-strengthening exercise for laryngeal elevation and pharyngeal contraction [6,12-14]. It is unclear whether THS provides enough muscle activity to be comparable to MM to enhance laryngeal elevation exercises.

The purpose of this study was to determine (1) whether THS training improves laryngeal elevation and (2) whether THS causes the intensity of muscle activity necessary to strengthen laryngeal elevation.

## Materials and methods

Study 1

Ten healthy participants aged between 60 and 79 years (74.0±3.9) were recruited. We excluded those with (1) a history of cerebrovascular disease, neurodegenerative disease, or head and neck cancer, (2) obvious dysphagia, such as cough associated with swallowing, or (3) an Eating Assessment Tool-10 (EAT-10) [15] score of 3 or higher. 

Participants were recruited through the center's administrator, who is a registered member of the Silver Human Resource Center and understands the purpose of this study. The administrator screened participants according to the inclusion and exclusion criteria. The principal investigator also reviewed the participant's medical history at the beginning of the study. 

The participants were given a written and verbal explanation of the study, and their consent was obtained.

The participants visited the speech therapy room of the hospital four times. The maximum tongue protrusion length (MTPL) [4] of each participant was measured upon their first visit. They were asked to swallow saliva with the tongue protruded to 50% of the MTPL. A tongue depressor showing the reference distance (50% of the MTPL) was given to each participant. The participants were instructed to perform THS while checking the tongue position with the tongue depressor each time. They were asked to repeat THS eight times per session, two sessions per day, and five days a week. They were also given a checklist to record whether they performed the exercise at home.

Upon the second visit, which was set two weeks after the first visit, the examiner checked whether the participants could perform the THS correctly. The checklist was also used to verify compliance with the home practice. The checklist consists of the date on which the training should be conducted, a column to indicate with a cross or a circle whether the training was conducted, and a remarks column. Then, they were instructed to perform THS with the tongue protruded to 67% of the MTPL. A tongue depressor showing the reference distance (67% of the MTPL) was given to each participant.

The third visit took place two weeks after the second visit. The participants underwent the same procedures as the second visit to confirm the appropriateness of THS and compliance with the exercise. Then, they were instructed to perform THS with the tongue protruded to 75% of the MTPL. A tongue depressor showing the reference distance (75% of the MTPL) was given to each participant. The fourth visit was arranged two weeks after the third, to confirm the appropriate performance of THS and compliance with the exercise.

**Figure 1:**
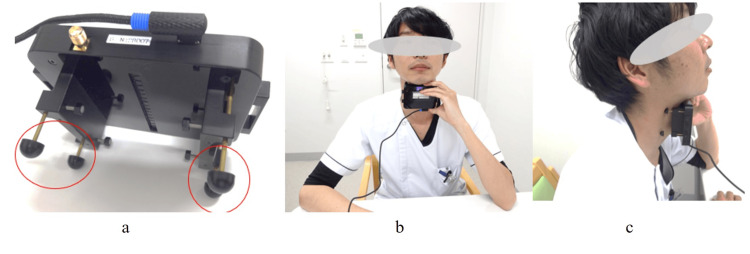
Measurement of laryngeal movement using a Nodomiru (OE-NDMR01®, Oisaka Electronic, Japan) (a) The central sensor array detects the movement of the larynx. The area indicated by the red circle is adjustable in length. (b) Frontal view of the participant and instrument. (c) Side view of the participant and instrument. The person in the photograph is a clinician demonstrating the procedure. Reference: [17]

The effects of the exercise were evaluated by the maximum tongue pressure and laryngeal elevation. The maximum tongue pressure was measured with a tongue pressure-measuring device (JMS, Hiroshima, Japan). This device measures maximum tongue pressure by placing a balloon-shaped probe in the mouth and pushing it back with the tongue. Measurements were done for each participant during each visit. A noninvasive laryngeal elevation measurement system (Nodomiru Application Version 1.07, Oisaka Electronic Equipment, Fukuyama, Japan) was used to measure the movements of laryngeal elevation (Figure 1a) [16,17].

The measurement system can be divided into two main parts. The first part was a measurement module that detects the skin shape near the larynx by placing 16 photoelectric distance sensors vertically aligned at 4-mm intervals relative to the front of the neck [16,17]. The second part was its software that takes the sensor information every 1/100th of a second. It identifies the larynx, analyzes, and displays the movement [16,17]. The Nodomiru can measure the anterior/posterior and upward/downward motions of the larynx. However, in previous papers [16,17], reproducibility has been verified only for the upward/downward motion. Thus, in the present study, only the upward/downward motion of the larynx was used.

The measurement procedure and posture of the Nodomiru followed [16]. Initially, the software was launched from a laptop computer and prepared for measurement. Then, the instrument's sensor array was placed vertically in the midline of the participant's neck, relative to the larynx. The participant held the instrument with one hand, and the position of the larynx was clarified by guiding the gaze slightly upward (Figure 1b, 1c). In addition, head and neck movements were suppressed by pressing the instrument against the neck, while the elbow rested on the desk. The principal investigator was the examiner of the laryngeal movements.

Two bolus types (i.e., saliva and 5 mL of water) were used. The participant was asked to accumulate "about the same amount of saliva as he would normally swallow." When saliva was held on the tongue, the participant signaled the examiner to start the measurement. For swallowing water, participants were asked to open their mouths, and 5 mL of water was placed in the anterior part of the mouth by using a syringe. Participants swallowed all the water in a single swallow, so that no water remained in the oral cavity after swallowing.

Swallowing methods consisted of two conditions: normal swallow (NS) and effortful swallow (ES). For ES, printed instructions were prepared in accordance with the study of Inamoto [2], and the maneuver was taught by a speech-language-hearing therapist specialized in dysphagia with 14 years of clinical experience. The participants were instructed to "imagine that there is a large lump of meat in your throat and swallow it as if you were pushing it in with a lot of force." After reading the instructions, the examiner demonstrated the steps as a model. Then, the participants were given 5-10 minutes of practice session.

The participants were asked to swallow the material three (during the second and third visits) or five times (during the first and fourth visits) in a total of four conditions: two bolus-type conditions and two swallowing methods. The effect of fatigue was reduced by randomizing the conditions of initiation.

For the maximum tongue pressure, a one-way analysis of variance (ANOVA) with a mixed model and a multiple-comparison test with Bonferroni were performed. The maximum tongue pressure of mean values was the dependent variable, the time of evaluation (first, second, third, and fourth) was the fixed factor, and individual patient and trial orders were the variable factors.

**Figure 2:**
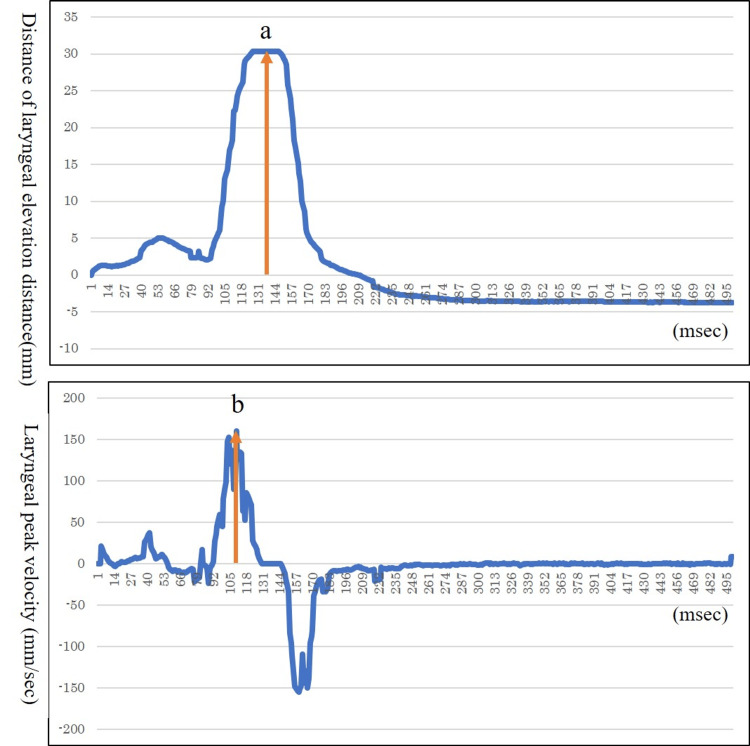
Indicators (distance of laryngeal elevation and laryngeal peak velocity) (a) Distance of laryngeal elevation. (b) Laryngeal peak velocity.

The analysis items for laryngeal elevation were peak velocity and distance, which were calculated according to Haji [16] (Figure 2). Each mean value (peak velocity and distance) of the first saliva/NS condition for each participant was normalized as 100. A one-way ANOVA was then performed with the peak velocity and distance of each condition as dependent variables, time of evaluation (first, second, third, and fourth) as the fixed factor, and individual patient and trial order as variable factors. IBM SPSS Statistics for Windows, Version 25.0 (Released 2017; IBM Corp., Armonk, New York, United States) was used for statistical analysis, and p<0.05 was judged significant.

Study 2

The iEMG values of THS and the Mendelsohn maneuver (MM), which are considered effective for strengthening laryngeal elevation, were compared to verify whether sufficient muscle activity was obtained in the submental muscle and thyrohyoid muscle region during THS.

Ten participants aged 69-79 years old (74.0±3.9), identical to those in study 1, were included. The iEMG values of the submental muscle region and thyrohyoid muscle region at NS, MM, and THS were compared at the fourth visit in study 1.

**Figure 3:**
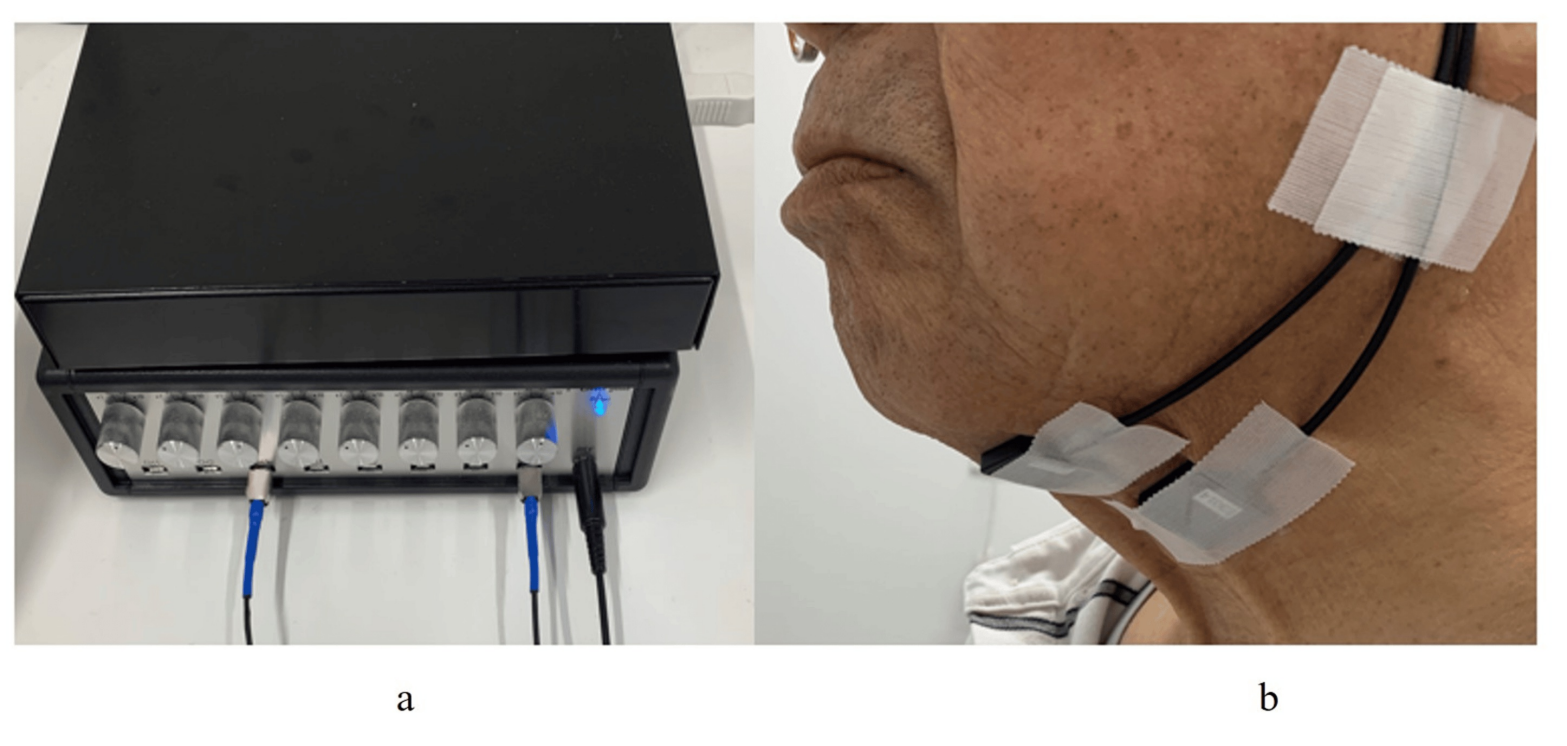
Example of an s-EMG device and surface electrode application (a) s-EMG device (P-EMG plus, Oisaka Electronics, Fukuyama, Japan). (b) Surface electrode application. The person in the photograph is not related to the participant. s-EMG: surface EMG

Surface EMG (s-EMG) was performed using a P-EMG plus (Oisaka Electronics, Fukuyama, Japan) (Figure 3a). The electrodes were myoelectric sensor extra small dry type FA-NSD2PADS for frequency analysis (17×20 mm, 10-mm distance between electrodes), and the ground electrodes were Blue Sensor M (40×34 mm, Ambu, Denmark). For FA-NSD2PADS, they were fixed with double-sided tape and surgical tape. The frequency ranged from 20 to 500 Hz, and the sampling frequency was 1 kHz.

The target muscles for the s-EMG were (1) the submental muscle region, which is bounded by the masticatory ridge and mandibular angle, and (2) the thyrohyoid muscle region on the side of the protuberantia mentalis. Because the preliminary experiments showed no left-right differences in bilateral muscle activity, the test muscle was unified on the left side in all cases (Figure 3b).

The participants were asked to perform NS, MM, and THS. For THS, the degree of tongue protrusion was set at 67% of the MTPL. For MM, they followed the instructions used by Inamoto [2]: when the larynx rises during swallowing, put pressure on the larynx and hold it up for three seconds. The participants practiced MM until a waveform depicted with a Nodomiru approximates a typical laryngeal elevation curve [17,18]. After the practice, participants took a break before the electrodes were applied.

Initially, muscle activity at rest was recorded. Then, the participant performed dry swallow to confirm that the muscle activity of the submental and thyrohyoid muscle region was recorded correctly. NS, THS, and MM were then performed five times each in a random order with breaks in between.

Measurements were taken for NS, MM, and THS with saliva held on the tongue. EMG during quiet breathing was recorded for approximately five seconds.

The examiner signaled the participant to swallow as instructed for each condition after five seconds. The measurement was completed after confirming that the larynx had descended. The mean muscle activity during any three seconds from the five seconds of quiet breathing was used as the baseline. Referring to Takahashi et al. [19], the first point at which the potential value exceeded ±2 SD relative to the potential value of the mean muscle activity of the submental muscles baseline was defined as the swallowing onset, whereas the last point at which the potential value exceeded ±2 SD was defined as the point of swallowing termination. Then, the muscle activity from the start of swallowing to the end of swallowing was considered the muscle activity for analysis. The amount of muscle activity was calculated by converting the EMGs under analysis into rectified waveforms, and iEMG and iEMG/s were calculated.

The iEMG and iEMG/s were normalized to the mean of five NSs for each individual as 100. The one-way ANOVA with a mixed model was performed with iEMG or iEMG/s as the dependent variable, swallowing method (NS, THS, and MM) as the fixed factor, and individual patient and trial order as variable factors, and multiple-comparison tests by Bonferroni were performed.

## Results

Study 1

All participants performed the THS procedure correctly each time. In addition, the checklist indicated that all participants complied with the frequency and number of times until the end of the procedure.

**Figure 4:**
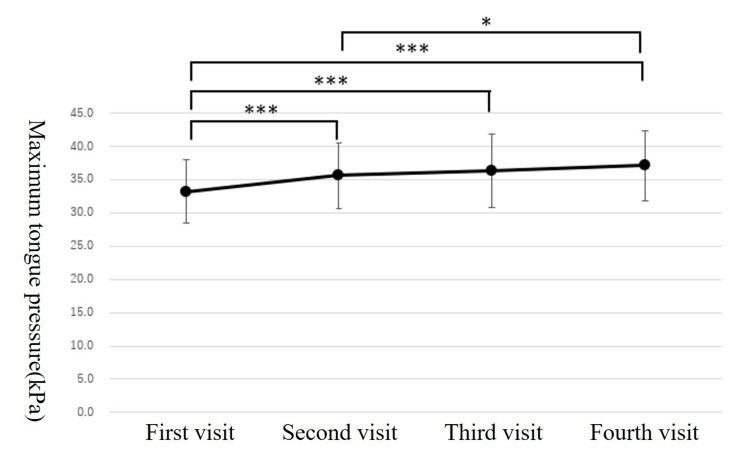
Change in the maximum tongue pressure over time The error bars indicate the standard deviation. One-way analysis of variance and multiple-comparison tests with mixed models. *: p<0.05; ***: p<0.001

The maximum tongue pressure of mean values in the first, second, third, and fourth visits were 33.2±5.6, 35.6±5.1, 36.4±5.5, and 37.1±5.3 kPa, respectively (Figure 4). The results of the ANOVA with mixed models showed significant differences (df=183, F=21.381, p<0.001). The results of Bonferroni's multiple-comparison test showed significant differences between the first and second (p<0.001), first and third (p<0.001), first and fourth (p<0.001), and second and fourth (p=0.027) visits, with gradually increasing maximum tongue pressure.

The results of ANOVA with mixed models showed a significant difference in peak velocity only in the water/ES condition (df=146.87, F=4.108, p=0.008). The results of the multiple-comparison tests were significantly greater in the fourth (p=0.041) than in the second (p=0.041) as well as in the fourth (p=0.025) than in the third visit (Table 1).

**Table 1:** Change in the peak velocity and distance for each condition P-values indicate the results of one-way analysis of variance with mixed models for each condition. *: p<0.05; **: p<0.01; NS: normal swallow; ES: effortful swallow; SD: standard deviation

		Peak velocity			Distance		
Conditions	Time	Average (SD)	P	Bonferroni	Average (SD)	P	Bonferroni
Saliva/NS	First	100.0 (16.1)	0.071		100.0 (13.8)	0.049	
	Second	108.9 (51.4)			110.3 (28.8)		
	Third	122.4 (66.6)			114.1 (44.5)		
	Fourth	106.1 (34.5)			110.2 (40.5)		
Water/NS	First	111.4 (43.0)	0.181		118.6 (31.8)	0.215	
	Second	131.7 (83.6)			119.5 (37.7)		
	Third	126.7 (72.5)			118.6 (31.2)		
	Fourth	113.4 (55.8)			114.9 (38.6)		
Saliva/ES	First	141.6 (92.6)	0.116		105.3 (29.4)	0.290	
	Second	123.9 (85.6)			114.7 (34.5)		
	Third	114.8 (65.8)			115.0 (30.9)		
	Fourth	136.0 (54.7)			111.9 (38.4)		
Water/ES	First	131.7 (91.8)	0.008		106.3 (36.0)	0.001
	Second	110.1 (87.0)		117.6 (37.6)		
	Third	108.1 (59.5)		125.1 (32.6)		
	Fourth	143.2 (83.7)			125.6 (35.3)		

The distance was significantly different only in the water/ES condition (df=146.566, F=5.707, p=0.001). In the multiple-comparison tests, the distance was significantly greater in the third (p=0.011) than in the first (p=0.011) and in the fourth (p=0.001) than in the first visit (Table 1).

Study 2

The iEMG/s of the submental muscle region was significantly different (df=138, F=5.340, p=0.006) as a result of one-way ANOVA with mixed models. The results of the multiple-comparison test showed that it was significantly greater in THS than in NS (p=0.006). The iEMG values of the submental muscle region were significantly different (df=138.013, F=12.739, p<0.001), which was significantly greater in THS than in NS (p=0.006) and in MM than in NS (p<0.001) (Table 2).

**Table 2:** Comparison of the muscle activities of the submental and thyrohyoid muscles in each method P-values indicate the results of one-way analysis of variance with mixed models for each indicator. **: p<0.01; ***: p<0.001; iEMG: integrated electromyography; NS: normal swallow; THS: tongue-hold swallow; MM: Mendelsohn maneuver; SD: standard deviation

Indicators	Methods	Submental muscle region			Thyrohyoid muscle region		
		Average (SD)	P	Bonferroni	Average (SD)	P	Bonferroni
iEMG/s	NS	100.0 (57.5)	0.006		100.0 (46.5)	0.102	
	THS	132.6 (83.7)			88.8 (29.6)		
	MM	124.1 (129.8)			112.6 (93.8)		
iEMG	NS	100.0 (86.9)	<0.001		100.0 (62.1)	<0.001	
	THS	204.9 (183.2)			138.2 (53.5)		
	MM	265.1 (356.2)			258.3 (214.7)		

No significant difference in iEMG/s was found for the thyrohyoid muscle region (df=138.0, F=2.322, p=0.102). The iEMG value for the thyrohyoid muscle was significantly different (df=138.003, F=25.326, p<0.001), which was significantly greater in MM than in NS (p<0.001) and in MM than in THS (p<0.001) (Table 2).

## Discussion

Improvement of the maximum tongue pressure

In the study by Lee et al. [10], older participants were randomly assigned into the THS group, tongue pressure resistance training group, and control group. After eight weeks of training, the THS group showed improvement in both anterior and posterior tongue pressure. Although the training period in the present study was shorter (six weeks) than their study, a significant improvement in the maximum tongue pressure was noted after six weeks.

Fujiu-Kurachi et al. [7] stated that restricted tongue movements during THS required a stronger-than-normal tongue retraction force during the oral phase of swallowing and that the magnitude of the load on the tongue was influenced by the degree of tongue protrusion. They also reported that the pressure generation time was longer at the posterior periphery of the palate during THS and that extra tongue movements occurred during swallowing with the inhibited anchoring function of the anterior tongue [7]. Thus, we speculated that THS led to an improvement in maximum tongue pressure because extra force is needed to move the tongue more than usual.

Effect on laryngeal elevation

In study 1, comparing the laryngeal elevation distance and peak velocity based on the value of the first visit, improvement was seen only in the laryngeal elevation distance in the water/ES condition.

In recent years, an increase in the tongue protrusion distance during THS was found to increase the amount of muscle activity of the supra- [19,20] and infrahyoid [19] muscles. Therefore, in the present study, three swallowing tasks (i.e., NA, MM, and THS) were included to compare muscle activity of the submental muscle region and thyrohyoid muscle region involved in the elevation of the hyoid bone and larynx. The results showed that the iEMG/s and iEMG values of the submental muscle region were significantly greater in THS than in NS, and no difference was found between MM and THS.

Fukuoka et al. [21] compared the root mean square of suprahyoid muscle activity in tongue-raising exercise with isometric contraction, tongue forward movement, head-raising exercise, and MM. They found that the tongue-raising exercise demonstrated the highest value; however, no significant differences were found among the other techniques. Considering these results, THS may not produce as much muscle activity as isometric tongue raising but may produce the same level of muscle activity in the submental muscles as the paratonsillar and head-raising exercises as well as MM. However, no significant difference in iEMG/s and iEMG of the thyrohyoid muscle region was found between NS and THS. In addition, the iEMG value was significantly greater in MM than in THS, indicating that the laryngeal elevation exercise should be combined with other training exercises to obtain sufficient muscle activity in both submental and thyrohyoid muscles when enhancing laryngeal elevation exercise in THS.

A large variation in the iEMG value of the submental muscle region was found in THS. The more the tongue protrudes, the more difficult it is for THS to initiate swallowing [7]. Thus, we speculated that the present results are caused by the differences in the effort-generating time to initiate swallowing. In this study, the tongue protrusion distance during THS was set at 67% of the MTPL, and EMG values were measured. This reflects the need to set the difficulty level by focusing not only on the tongue protrusion distance but also on the time required to initiate swallowing.

Robbins et al. [22] found that eight weeks of lingual exercise produced significant increases in tongue pressure and swallowing pressure. Specifically, the most significant change was noted in the 3-mL effort swallow of several conditions (3 mL of liquid, 10 mL of liquid, 3 mL of semisolid, and 3-mL effort swallow). The improvement in tongue and swallowing pressure possibly contributed to the improvement in laryngeal elevation distance in the water/ES condition.

THS has the potential to increase the posterior pharyngeal wall contraction and strengthen not only the movement of tongue retraction but also the laryngeal elevation. Further studies on the number of repetitions, frequency, and level of difficulty of THS will enable us to develop a comprehensive training program for THS in the future.

Limitations of this study

This study did not have a control group; thus, the changes in the participants after six weeks could not be attributed to training. In addition, the participants were limited to older men and healthy individuals with a small sample size; hence, careful discussion is necessary when interpreting the results. The results of this study should be regarded as preliminary findings. Further studies expanding the participant population by including women and patients with dysphagia are warranted.

## Conclusions

After THS training, the maximum tongue pressure and laryngeal elevation distance in the water/ES condition improved in healthy older men. In addition, THS and MM produced similar muscle activity in the submental muscle region but not in the thyrohyoid muscle region. Although THS may be effective in increasing the posterior pharyngeal wall contraction and tongue retraction, combining THS with other exercises should be considered for the purpose of enhancing laryngeal elevation.
